# Leukocyte Gene Expression in Patients with Medication Refractory Depression before and after Treatment with ECT or Isoflurane Anesthesia: A Pilot Study

**DOI:** 10.1155/2014/582380

**Published:** 2014-04-13

**Authors:** E. Iacob, S. C. Tadler, K. C. Light, H. R. Weeks, K. W. Smith, A. T. White, R. W. Hughen, T. A. VanHaitsma, L. A. Bushnell, A. R. Light

**Affiliations:** ^1^Department of Anesthesiology, University of Utah Health Sciences Center, Salt Lake City, UT, USA; ^2^Neuroscience Program, University of Utah Health Sciences Center, Salt Lake City, UT, USA; ^3^Department of Psychiatry, University of Utah, Salt Lake City, UT, USA; ^4^Department of Exercise and Sport Science, University of Utah, Salt Lake City, UT, USA

## Abstract

*Objective*. To evaluate leukocyte gene expression for 9 selected genes (mRNAs) as biological markers in patients with medication refractory depression before and after treatment with ECT or isoflurane anesthesia (ISO). *Methods*. In a substudy of a nonrandomized open-label trial comparing effects of ECT to ISO therapy, blood samples were obtained before and after treatment from 22 patients with refractory depression, and leukocyte mRNA was assessed by quantitative PCR. Patients' mRNAs were also compared to 17 healthy controls. * Results*. Relative to controls, patients before treatment showed significantly higher IL10 and DBI and lower ADRA2A and ASIC3 mRNA (*P* < 0.025). Both ECT and ISO induced significant decreases after treatment in 4 genes: IL10, NR3C1, DRD4, and Sult1A1. After treatment, patients' DBI, ASIC3, and ADRA2A mRNA remained dysregulated. *Conclusion*. Significant differences from controls and/or significant changes after ECT or ISO treatment were observed for 7 of the 9 mRNAs studied. Decreased expression of 4 genes after effective treatment with either ECT or ISO suggests possible overlap of underlying mechanisms. Three genes showing dysregulation before and after treatment may be trait-like biomarkers of medication refractory depression. Gene expression for these patients has the potential to facilitate diagnosis, clarify pathophysiology, and identify potential biomarkers for treatment effects.

## 1. Introduction

Blood-based biomarkers are important to the understanding, diagnosis, and treatment of medical disorders. They can be especially helpful when the disorder is poorly understood, complex, or where the differentiation of subgroups may have important implications for treatment and long-term outcomes. In the case of depressive disorders (DD), one subgroup where biomarkers would be especially useful consists of those patients who are refractory to pharmacologic antidepressant therapies. Previous studies have suggested that gene expression (mRNA) on white blood cells may provide useful biomarkers for patients with DD, including those with refractory depression [1, 2].

Patients with medication refractory depression (typically defined by failure to achieve remission with two or more trials of different antidepressant regimens) represent almost one-third of all patients with DD [3]. There are relatively few treatment options for these patients. Electroconvulsive therapy (ECT) is widely acknowledged to be the most effective option, achieving remission in 55–90% of the cases, even among patients who were previously refractory [4, 5]. Despite its safety and efficacy [6], many millions of patients with refractory DD elect not to receive ECT due, in large part, to misperceptions about this treatment being painful or traumatic or to concerns regarding associated adverse cognitive effects, most of which are, however, temporary [7, 8]. This has encouraged efforts to find alternative therapies to ECT that are effective in treating medication refractory DD, in addition to having greater social acceptability and minimal adverse effects on memory and cognitive function. One alternative therapy originally proposed two decades ago is high dose inhalation anesthesia using isoflurane (High ISO). Like ECT, High ISO induces a brief state of electrocortical quiescence (burst suppression, BS), but does so without inducing the ECT seizure or the adverse memory and cognitive symptoms. Langer et al. first reported that a series of 6 treatments with High ISO had similar efficacy to 6 ECT sessions in reducing depressive symptoms without causing memory loss [9, 10]. Further research on the antidepressant effects of ISO or sevoflurane yielded mixed results. Positive antidepressant effects of High ISO were reported by Carl et al. [11] and Engelhardt et al. [12]. In contrast, Greenberg et al. [13] reported little improvement with High ISO treatments in 6 elderly depressed patients, and García-Toro et al. [14] found only limited improvement after 4 prolonged treatments of low dose sevoflurane (24% reduction in depressive symptoms).

There was essentially a cessation of interest in this potential alternative treatment until recently, when our research group completed an open-label study comparing ECT versus High ISO inducing BS [15]. We suggested that the previous unsuccessful studies used too few treatments when, indeed, even the gold standard intervention, ECT, requires 8–12 treatments for maximal effectiveness. Also, we hypothesized that anesthetic treatment at doses that are too low to produce strong cortical BS (like the sevoflurane dose used by García-Toro et al. [14]) would have minimal antidepressant effects. Thus, we compared responses of 8 patients with medication refractory depression treated with 10 sessions of High ISO with confirmed BS to 20 patients treated with bifrontal ECT. Our findings showed that High ISO led to an antidepressant response defined as a 50% reduction on the Hamilton rating scale of depression (HRSD score) in 75% of patients and an identical rate of full remission of depressive symptoms as ECT (50%). Also, using standardized neuropsychological tests of memory and processing speed, ECT led to temporary declines in cognitive function and more lasting deficits in autobiographical memory, but 10 treatments with high ISO did not.

As a pilot substudy ancillary to our open-label study [15] comparing High ISO/BS versus ECT treatment, we sought to evaluate the utility of using leukocyte gene expression as a biomarker of disease for this medication refractory group of DD patients. Our aims were to improve our understanding of the pathophysiology of refractory DD and to assess whether any changes induced by the two different treatments were similar or different. Thus, we obtained blood samples from 22 of the patients with medication refractory DD for determination of leukocyte gene expression at both Pretreatment and Post-treatment (24–48 hours after the final treatment session). We also obtained blood samples from 17 healthy, nondepressed controls. We examined a profile of 9 mRNAs, consisting of immune marker IL10; adrenergic receptor ADRA2A; hypothalamic-pituitary-adrenal (HPA) axis associated receptors including the glucocorticoid receptor (NR3C1), dopaminergic receptor-4 (DRD4), and diazepam-binding inhibitor (DBI, aka the GABA receptor modulator). We also included the sulfotransferase family cytosolic 1a (Sult1A1) involved in the metabolism of monoamines and drugs. For a transcription gene linked to multiple levels of cellular regulation and metabolism, we included VEGFA, previously shown to differ in patients with major depressive disorder (MDD) compared to controls [16]. Finally, we included two ion channel receptors of the acid sensing ion channel (ASIC) family. These ion channel genes were selected based on prior research in our laboratory showing associations with chronic fatigue syndrome and fibromyalgia [17, 18], animal models showing associations with depression [19], and mRNA in postmortem tissue [20]. Among the behavioral functions relevant to MDD linked to ASIC pathways are anxiety, aggression, and pain [21].

Our objectives for this small pilot study were to determine (1) whether patients with refractory DD would differ from controls in expression of selected target genes prior to treatment, (2) whether 10 treatment sessions of High ISO and 8–12 sessions of ECT would induce similar or different changes in expression of these target genes, and (3) whether some genes would show persistent differences in these patients from levels in controls even after treatment, indicating that these may be trait-like biomarkers of refractory DD.

## 2. Materials and Methods

The study protocol was approved by the University of Utah Institutional Review Board (IRB) and all subjects gave written consent prior to participation.

### 2.1. Subjects

Subjects in the DD group included 22 patients (14F/8M, mean age 40.00 ± 2.44) with medication refractory depression who had been referred for consultation for possible electroconvulsive therapy (ECT) to the University of Utah Neuropsychiatric Institute (UNI). Patients were diagnosed according to the criteria in the* Diagnostic and Statistical Manual of Mental Disorders, Fourth Edition *(DSM-IV) [22]. Seventeen met criteria for major depressive disorder (MDD) and 5 for with bipolar disorder (BPD), with current phase being moderate to severe depression. Exclusion criteria have been described previously [15]. Demographic and clinical parameters for patients are listed in Table 1.

These patients represent the majority (78.6%) of the medication refractory DD participants from a nonrandomized, open-label study comparing the cognitive side effects and treatment efficacy of High ISO anesthesia versus ECT [15]. Blood samples were collected before the start of High ISO or ECT treatments (pretreatment) and 24 hours after the last treatment session (posttreatment). The original study included 28 patients: 20 patients who elected to receive ECT and 8 patients who elected to receive High ISO. Of the 20 ECT patients, 5 patients received treatment before the gene expression ancillary study began, and 1 patient had a blood sample that did not meet quality standards (not enough total RNA). This left 14 ECT subjects that were included in the analysis. In our ECT sample, one patient had a pretreatment blood sample but no posttreatment sample. All 8 patients receiving ISO had acceptable blood samples at both pretreatment and posttreatment.

The 14 ECT patients received 8–12 treatments, while the 8 ISO patients received 10 treatments [9, 10, 15]. Clinical assessment at pretreatment and posttreatment was carried out using the Hamilton rating scale for depression (HRSD-24), a structured clinician-administered interview [23, 24]. Depression severity was also assessed prior to each treatment session using the quick inventory of depressive symptomatology (QIDS) self-report. The QIDS self-report has been found to have concurrent validity with the HRSD [25]. Patients with the most severe depression more frequently elected ECT versus ISO; thus, the pretreatment HRSD mean value was higher in the ECT versus the ISO group. For all DD participants, the decision was made to maintain pretreatment antidepressant, antipsychotic, and other psychoactive medications throughout the study. Specific medications are noted in Table 1. Of note, some patients were asked to stop anticonvulsant medications prior to treatment. Otherwise, medication regimens were not altered proximate to treatment and testing.

Controls included 17 subjects (11F/6M, mean age 47.53 ± 3.1). These participants provided medical history information including all current medications and completed the QIDS-SR. None were taking antidepressants or had current symptoms or history of depression.

### 2.2. mRNA Extraction and Analysis

All blood processing and analyses were performed by personnel being blinded to the subjects' group. Blood was collected in EDTA tubes and kept on ice, and within 15 minutes after collection was centrifuged at 3200 rpm (1315 ×g-Clay Adams Compact II Centrifuge) for 12 minutes. The plasma was removed, and the white layer carefully collected in RLT+*β*-ME (Qiagen, Valencia, CA), then quickly frozen using a methanol-dry ice slurry and stored at −80°C. RNA was extracted using RNeasy mini kits (Qiagen, Valencia, CA) and treated with RNase-free DNase-I (Qiagen, Valencia, CA). Immediately following extraction, RNA was converted to a cDNA library using the ABI High Capacity cDNA Archive Kit (Applied Biosystems/Life Technologies, Inc., Foster City, CA), and then treated with RNase-H (Epicentre Biotechnologies, Madison, WI). The cDNA samples were stored at −20°C until analysis. RNA integrity was assessed with a Bioanalyzer and consistently found to have RIN values (RNA integrity numbers) greater than 9. The cycle counts for the control gene, TF2B, averaged 21.78 ± 1.67 (SD) for control subjects and 22.32 ± 2.09 (SD) for patients, Student's *t*-test *P* = 0.12. We selected TF2B as the preferred control gene and have verified that TF2B qPCR counts do not change in freshly harvested separated human leukocytes under a variety of conditions, when RNA is processed as described here.

The cDNA libraries were analyzed using the ABI quantitative, real-time PCR system on the ABI Prism 7900 Sequence Detection System (SDS) 2.4.1 (Applied Biosystems, Inc., Foster City, CA), using ABI TaqMan Master Mix (Applied Biosystems, Inc., Foster City, CA). Master mix/primer plus primer/probe solutions and template solutions were separately loaded onto 96-well preplates. Then 384-well plates were robotically loaded and mixed from the 96-well plates. Each targeted gene was examined in duplicate, with TF2B standards being run in quadruplicate. Additional control samples containing no template were also run. Primer probes for the 9 genes were obtained from TaqMan Gene Expression Assays (Applied Biosystems, Inc., Foster City, CA) and were as follows: Adrenergic A2A—Hs00265081_s1; ASIC1—Hs00241630_m1; ASIC3—Hs00245097_m1; diazepam binding inhibitor—Hs00220950_m1; dopamine receptor 4—Hs00609526_m1; glucocorticoid receptor NR3C1—Hs01005217_m1; IL10—Hs00174086_m1; sulfotransferase 1A1—Hs00738644_m1; vascular endothelial growth factor A—Hs99999070_m1; and control primer probe TF2B—Hs00155321_m1q. PCR data was processed using the SDS2 program from Applied Biosystems with count values for genes computed in the curve log-linear using a standard 0.2 threshold. Gene expression amounts were determined using the 2^−Δ*T*^ method, where Δ*T* is the count difference of the candidate gene from TF2B.

### 2.3. Statistics

All statistical tests were performed using STATA ver.12 statistical software. Demographic variables were compared using Student's *t*-test for metric variables and Pearson *χ* ^2^ for nominal variables. HRSD and QIDS data were analyzed with repeated measures ANCOVAs (2 × 2 treatment group × time for HRSD and 2 × 8 treatment group × time for QIDS) with age as a covariate, to compare ECT and ISO treatment groups at pretreatment and posttreatment and to examine changes in depression severity between those time points. Age was retained in the final model only if it approached significance (*P* < 0.10). Gene expression values were log transformed in order to satisfy normality and equality of variance assumptions. For each gene, repeated measures ANCOVAs (2 × 2 treatment group × time) with age as a covariate were used to compare ECT and ISO treatment groups at pretreatment and posttreatment and to examine changes in gene expression between those two time points. One of the 14 ECT patients who did complete the HRSD and QIDS assessments did not have a posttreatment blood draw. For the repeated measures gene expression analyses where data were missing, the more conservative decision was made to use the reduced sample size rather than use regression-based methods to interpolate the missing values. When no significant main effect of treatment group or group × time interaction was obtained but the effect of time was significant, data from both the ECT and ISO groups were combined into a single DD group for comparison of differences between pretreatment and posttreatment levels. As a second level of analysis, these same pretreatment and posttreatment levels from the DD group were compared to the levels obtained from controls. Cohen's *f* effect sizes were computed for ANOVA models with 0.02, 0.15, and 0.35 interpreted as small, moderate, and large effect sizes, respectively. Statistical values are reported as one-tailed tests for differences involving* a priori *hypotheses (differences between patients and controls and changes from pretreatment to posttreatment), because in a pilot study, there is a greater consequence for Type II versus Type I error. All other tests were two-tailed. As a correction for multiple comparisons, alpha level for analyses involving all DD patients versus controls was set at *P* < 0.025 rather than *P* < 0.05.

## 3. Results

### 3.1. Depression Severity Decreases following ECT and ISO

**Figure 1:**
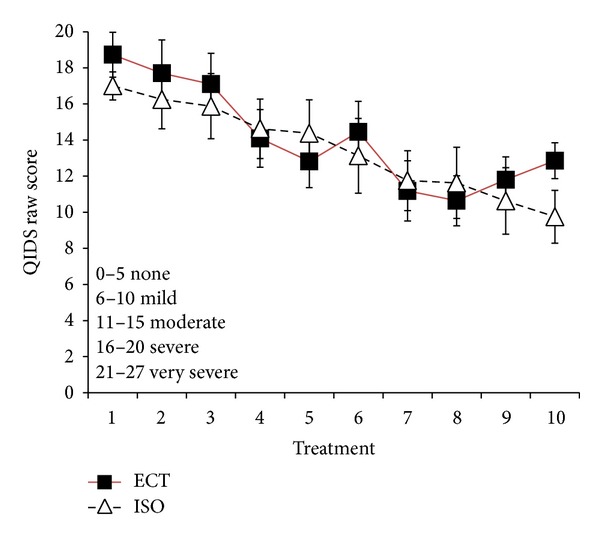
ECT and ISO display progressive improvements on quick inventory of depression scale (QIDS) self-report taken prior to each treatment. ECT patients are in solid squares and ISO patients in open triangles. Both treatment groups display progressive improvement over the 10 treatments. As described in the demographics table, all ISO patients received 10 treatments and completed their QIDS-SR prior to each treatment. Conversely, 11/14 ECT patients completed their QIDS. For data at treatments 9 and 10, there were 10 and 7 patients, respectively, in the ECT group. The slight upturn at treatments 9 and 10 in the ECT group is the result of the more severely depressed patients requiring those final treatments.

Consistent with our prior report from the full patient sample [15], in this subsample, the ECT patients displayed significantly higher pretreatment HRSD depression severity compared to the ISO group (*t* = 3.90, *P* < 0.01). Both ECT patients and ISO patients displayed significant HRSD improvement at posttreatment relative to pretreatment (paired *t* = 7.50, *P* < 0.001 for ECT; paired *t* = 3.61, *P* < 0.01 for ISO). Based on self-reported depressive symptoms using QIDS scores obtained prior to each treatment session, patients in the ECT and ISO groups did not differ at any time, and both groups showed progressive improvement during the treatment sessions (see Figure 1). Since all patients received at least 8 treatment sessions, repeated measures analysis based on the first 8 QIDS scores showed no significant differences between treatment groups (*P* = 0.972) and no significant group × time interaction (*P* = 0.6084); however, the main effect of time on QIDS scores was significant (*F*(1,173) = 13.11, *P* < 0.00001; Cohen's *f* effect size = 0.4376). Subsequent contrasts indicated a marginally significant QIDS score decrease from session 1 to session 3 (*P* = 0.064) and significant decreases from session 1 to sessions 4–8 (*P* < 0.0003–*P* < 0.00001).

### 3.2. Gene Expression Differences in ISO and ECT Groups and Combined DD Group versus Controls

Initial ANCOVAs revealed no significant pretreatment mRNA differences between the ECT and ISO treated patients (all* P*'s > 0.19; see Table 2). When the pretreatment mRNA levels of the combined DD patients were compared to levels shown by controls, significant differences were obtained for 4 genes: the adrenergic receptor ADR2A (*F* = 23.55, *P* = 0.0013, and Cohen's *f* = 0.437), the acid sensing ion channel ASIC3 (*F* = 10.39, *P* = 0.003, and Cohen's *f* = 0.511), the GABA receptor modulator DBI (*F* = 15.29, *P* < 0.0004, and Cohen's *f* = 0.613), and the cytokine IL10 (*F*(1,36) = 5.77, *P* = 0.022, and Cohen's *f* = 0.354). Subsequent comparisons among means showed that the separate ECT and ISO patient subgroups had significantly decreased expression of ADRA2A and ASIC3 and significantly increased expression of DBI and IL10 at pretreatment compared to controls (see Table 2). The consistency of these findings in both treatment groups strengthened our confidence in the mRNA similarity of the two patient groups prior to receiving treatment.

### 3.3. Gene Expression in ISO versus ECT Groups at Pretreatment versus Posttreatment

In our second set of analyses where posttreatment and pretreatment levels were compared, the ANCOVAs revealed no significant main effect of treatment group or group × time interactions for any of the 9 genes (see Table 3). Thus, there no mRNA differences between the ECT and ISO groups either at posttreatment or at pretreatment. However, there were significant main effects of time (pre- versus posttreatment differences) for 4 genes: DRD4 (*F*(1,19) = 3.72, *P* = 0.035, Cohen's *f* = 0.137), IL10 (*F* = 4.57, *P* = 0.025, and Cohen's *f* = 0.111), NR3C1 (*F* = 21.63, *P* < 0.001, and Cohen's *f* = 0.379), and Sult1A1 (*F* = 3.55, *P* = 0.041, and Cohen's *f* = 0.169). Subsequent contrasts between paired means showed that all these 4 genes decreased significantly at posttreatment relative to pretreatment; note that both the ISO and ECT groups show these same treatment-related changes in expression of these 4 genes (see Table 3). These findings indicate that changes in expression of some of the genes under study did occur between pretreatment and posttreatment and these changes were not unique to either ECT or ISO but similar in both treatment groups. For expression of one gene, VEGFA, there was a marginal trend for a group × time interaction (*F* = 2.49, *P* = 0.067, Cohen's *f* = 0.194), suggestive of a possible exception to the general pattern of similar effects of the two treatments.

It is also notable that of the 4 genes that differed in DD patients versus controls, 3 were not significantly changed from pretreatment to posttreatment: ADR2A, ASIC3, and DBI. When posttreatment levels of these genes were then compared to mRNA levels of controls, our comparisons revealed that similar to pretreatment, DD patients continued to have significantly higher DBI mRNA (5.48*E* − 03 versus 3.96*E* − 03, *F* = 16.00, *P* < 0.001, and Cohen's *f* = 0.664), lower ADRA2A (2.95*E* − 03 versus 5.26*E* − 03, *F* = 10.66, *P* = 0.014, and Cohen's *f* = 0.170), and marginally lower ASIC3 mRNA (6.72*E* − 03 versus 8.32*E* − 03, *F* = 3.19, *P* = 0.08, and Cohen's *f* = 0.246). In contrast, at posttreatment, IL10 was no longer increased in DD patients versus controls (*F* = 2.35, *P* = 0.136, and Cohen's *f* = 0.199). Additionally, patients displayed decreased glucocorticoid receptor NR3C1 expression compared to controls at posttreatment (5.20*E* − 01 versus 7.90*E* − 01; *F* = 8.70, *P* = 0.006, and Cohen's *f* = 0.476), although expression of this gene was not significantly increased in DD patients at pretreatment.

## 4. Discussion

### 4.1. Effects of Isoflurane Anesthesia and ECT Treatment on Gene Expression

There are two principal findings of this pilot gene expression study. (1) Four of the 9 genes studied showed significantly decreased expression at posttreatment compared to pretreatment, and all these 4 genes changed similarly after both the ECT and ISO treatment conditions. This finding suggests that these 4 physiological pathways were altered in the same way by both of these effective antidepressant treatment regimens, and that these changes may reflect common mechanisms underlying the beneficial symptom improvement. The genes in question were the dopamine receptor DRD4, the cytokine IL10, the glucocorticoid receptor NR3C1, and the enzyme Sult1A1. Of these 4 genes, only IL10 was also significantly elevated at pretreatment in these medication refractory DD patients compared to controls. (2) Compared to healthy control participants, DD patients had increased GABA receptor modulator DBI and decreased adrenergic receptor ADRA2A and ASIC3 expression at pretreatment that remained dysregulated after treatment, despite depressive symptom improvement as reflected in HRSD and QIDS scores. Although our sample sizes are quite small and thus these findings remain preliminary, it was reassuring to see that when the ECT and ISO patient subgroups were examined separately; these differences from controls were significant in both groups (Table 2). Thus, our preliminary interpretation is that these 3 mRNAs may be trait-like biomarkers of medication refractory depression and may indicate physiological pathways that could potentially be linked to subsequent susceptibility to relapse.

Despite over 70 years of clinical use, the precise biological mechanisms underlying the antidepressant effects of ECT are not fully understood. Changes in cerebral blood flow, electrical activity of the cortex, monoamine effects, inflammation, growth factors, and neurogenesis have all been proposed [26–30]. Many of these elements are combined in the “anticonvulsant theory” of ECT's effects. This theory asserts that with each successive ECT treatment and each induction of a seizure, there are secondary changes that occur to raise the seizure threshold and minimize the seizures, including long-lasting changes in seizure threshold, cerebral blood flow, and the patterns of electrical activity of the cortex, as well as changes in the release of neurotransmitters and neuropeptides [31]. One strength of this theory is that it accounts for why multiple ECT treatment sessions are necessary. To date, however, there are no compelling data confirming or invalidating the correctness of these hypothetical mechanisms. However, recent studies have shown that preconditioning with isoflurane and other anesthetics has neuroprotective effects against later ischemic events that involve multiple mechanisms which appear to overlap with components of this theory of ECT's effects [32].

The potentially important role of cortical EEG burst suppression in producing antidepressant effects of ECT or anesthesia was first proposed by Langer et al. as they justified their pioneering investigation of isoflurane treatments as an alternative to ECT [9, 10]. Their rationale was that the reduction in electrocortical activity was the feature shared by High ISO and ECT. Kranaster et al. reported that the BS index correlated with seizure duration in ECT treatments and thus would be a useful predictor of antidepressant effectiveness [33]. This interpretation has not previously been studied in any rigorous way in humans but has been reinforced by recent findings using animal models of depression. Research in rats by Murrell et al. indicated that isoflurane had greater potency to induce EEG BS than two related anesthetic drugs, sevoflurane and desflurane, and that another inhaled anesthetic, halothane, did not induce BS [34]. Using the learned helplessness behavioral model of depression in rats, Wang and colleagues found that 2 hours of isoflurane at 2% prevented this depressive behavior, while anesthetic-naive or halothane-treated rats showed no preventive antidepressant effects [35].

Whether cortical burst suppression is the underlying mechanism for the antidepressant effects of ECT or ISO anesthesia is still an open and important research question to address. The findings of the present study, however, do support the hypothesis that ECT and ISO anesthesia treatments may have similar effects on a number of neural and immune pathways that could contribute to the reduction in depressive symptoms observed in both treatment conditions. All four genes that were significantly decreased after ECT were similarly decreased after high dose ISO treatments. Decreases in expression of the glucocorticoid and dopamine receptors NR3C1 and DRD4 and in the expression of Sult1A1, an enzyme that promotes the breakdown of neurotransmitters, neuropeptides, and hormones, might be predicted by the anticonvulsant theory of ECT's effects. Also, decreases in expression of several cytokines (but not IL10) have previously been reported following a positive response to antidepressant treatment with escitalopram or nortriptyline in the large GENDEP investigation [1]. It should be noted that in the current study, we examined gene expression changes 24 hours after the last treatment and therefore patients had hereto received 8–12 treatments over the span of 2-3 weeks. Therefore, changes may represent an acute response (from the treatment 1-2 days before), as well as a response to prolonged exposure to treatment. Also, expression of one gene, VEGFA, showed a trend for a differential effect of ECT versus ISO treatment (*P* = 0.067), an effect that might have been significant if our sample size had been larger. Future research with larger, randomized samples and larger gene arrays are needed to verify whether ECT and ISO effects on depression primarily involve the same or different physiological pathways.

### 4.2. Functions of Genes That Show Treatment-Related Changes

We observed that patients had elevated levels of the anti-inflammatory cytokine IL10 at pretreatment that were significantly reduced at posttreatment. Cytokines are important signaling molecules produced by the brain and immune cells to induce inflammation in response to stress and foreign pathogens. A wealth of previous research has implicated the immune dysregulation in DD patients including elevated levels of specific cytokines such as IL10, IL6, and TNF*α* [36]. Elevated levels of IL10 mRNA are consistent with previous gene expression studies in patients during a depressive episode [37], although a meta-analysis suggests that protein concentrations of IL10 do not differ between patients with depression and controls [38]. Previous studies have shown no immediate change in IL10 protein levels within 24 hours following ECT treatments, although levels did decrease gradually over time with multiple ECT treatments [39, 40]. In terms of mRNA, Belzeaux et al. found that IL10 levels that were elevated prior to medical treatment did decrease and were no longer significantly higher than controls after 8 weeks [41]. We did not evaluate another key cytokine, IL6, in this study; however, we have previously reported that IL6 and IL10 expression increased in female patients with active medication refractory depression [42]. IL6 mRNA decreases following medical treatment were also linked to being a responder in the GENDEP study [1]. These findings suggest that immune dysregulation may be related to the etiology of depressive symptoms. Expansion of immune markers to include protein as well as mRNA should be considered for future studies.

We also observed consistent treatment-related decreases in expression of the glucocorticoid receptor, NR3C1. When each individual patient's response was examined, nearly all patients (85% of ISO and 90% of ECT) displayed decreased levels of NR3C1 24–48 hours following the last treatment. The glucocorticoid pathway, which is involved in the immune response, particularly in inhibiting inflammatory responses, has previously been shown to be dysregulated in some patients with depression [43]. Animal studies have found that electroconvulsive stimulation (ECS) can normalize stress-induced decreases in brain hippocampal glucocorticoid receptor mRNA [44]. Furthermore, ECT has been shown to normalize cortisol response in a small study of 7 patients successfully treated with ECT [45]. The finding that decreased NR3C1 mRNA was present in both treatment groups suggests ISO treatment may also affect this glucocorticoid pathway, in ways similar to ECT.

The dopamine receptor DRD4 showed decreases in all DD patients from pretreatment to posttreatment though not versus controls. Dopamine plays an important role in reward-seeking and motivation, the disruption of which can lead to anhedonia. Decreases in expression of the DRD4 receptor may be compensatory changes responding to an increased release of dopamine itself. This is potentially important, given that dopamine mediates some bidirectional communication between the central nervous system and immune function [46]. In preclinical models, ECS has led to enhanced dopaminergic function as well as upregulation of D1 and D3 receptor binding, while decreased D2 receptor binding has been observed in brain regions of humans [47]. In rat studies, isoflurane anesthesia has also been shown to increase levels of dopamine [48] and modulate subsequent dopamine release associated with psychotropic medications [49]. Excess dopamine release and receptor activation may lead to the decreases in DRD4 mRNA that were observed. Prior gene expression studies found low pretreatment DRD4 mRNA levels in medication-responsive depressed patients that normalized to control levels following successful treatment with the antidepressant paroxetine [50]. In other studies, DRD4 levels in postmortem brain tissue from MDD and schizophrenia patients were found to be elevated [51, 52]. Variations in peripheral and postmortem levels of DRD3 and dopamine transporter have also been observed, suggesting broad dysregulation of dopaminergic pathways in depressive disorders [53, 54].

For the enzyme Sult1A1, DD patients displayed pretreatment levels similar to those in healthy controls but still had significant decreases at posttreatment. Sult1A1 is responsible for the inactivation and ultimate metabolism of a number of compounds, including hormones, catecholamines, and drugs [55]. Thus, decreases in Sult1A1 mRNA and the enzyme product may lead to increased availability of these important signaling molecules and medications. Though research has shown that genetic variants of Sult1A1 can increase risk of breast or colorectal cancer, few studies have found differences in depressive disorders [55, 56]. One study by Marazziti et al. found variability among different mood disorders in platelet protein levels of phenosulfotransferase (PST), which is coded by Sult1A1; these variations included increases in patients with obsessive compulsive disorder and mania, decreases in patients with MDD or migraine, and no differences with BPD relative to controls [57]. No prior study, however, has examined patients with medication refractory DD, who might be expected to differ from medication responders given the effect of Sult1 A1 on drug metabolism.

### 4.3. Functions of Genes Differing Consistently between Patients and Controls

In contrast to the genes discussed above, three genes showed differential expression in the current sample of patients with refractory depression versus controls that was not altered by treatment. The first was the diazepam binding inhibitor (DBI), a gene that is a negative allosteric modulator of GABA_A_ receptors, which has previously been implicated in both depression and anxiety [58–60]. We found DBI mRNA levels to be significantly increased in the DD group at pretreatment. This is in line with previous studies that found increased levels of DBI protein in cerebral spinal fluid (CSF) of drug-free depressed patients [59, 61]. Interestingly, other research showed a positive correlation between levels of DBI and the corticotrophin releasing hormone (CRH) in depressed patients, gamblers, and controls [62]. Since GABA receptors inhibit CRH producing cells, it is possible that elevated levels of DBI lead to increased levels of CRH and ultimately elevated cortisol, a common signature of HPA dysfunction in many depressed patients [63, 64]. Finally, peptide fragments of DBI decrease the effect of benzodiazepines on proinflammatory cytokines following immune challenge, suggesting that increased levels of DBI may interfere with the anxiolytic action of benzodiazepines and their effect in HPA regulation [65]. This finding and our own observations support the possibility that increased DBI may be a stable biomarker for treatment-refractory depression.

We also found that mRNA gene expression for the adrenergic receptor ADRA2A was decreased at both pretreatment and posttreatment in depressed patients versus controls, even after controlling for an association with age. This receptor's primary neurotransmitter, norepinephrine (NE), has roles in acute and chronic stress, as well as in mediating inflammation [66–68], and is one of the primary targets of NRI and SNRI antidepressants. Previous human and animal studies have found that depression is associated with elevated levels of the ADRA2A receptor protein and binding sites [69–72], though some studies suggest no change or even a decrease in binding [73, 74]. Since mRNA levels do not necessarily correlate with protein production, our finding of decreased ADRA2A in medication refractory DD could be indicative of compensation for high protein expression. Future research that concurrently determines both ADRA2A mRNA and protein levels in refractory DD patients is needed to confirm this explanation.

Finally, we found consistently decreased expression of ASIC3, a member of the acid-sensing ion-channel family. Because of the high comorbidity of depression with pain symptoms and the fact that antidepressants can help with pain symptoms, it is likely that receptors that mediate the sensations of pain and fatigue may be dysregulated in depression [75, 76]. In this study, we examined two ATP-responsive receptors from the acid-sensing ion-channel family (ASIC1 and ASIC3). In the case of the ASIC1a receptor, ASIC1a knock-out mice and wild-type mice treated with ASIC1a inhibitors both show antidepressant and anxiolytic behavior [19, 77]. Previous results from our research group showed that ASIC3 receptor expression, although normal at baseline, was increased within 30 minutes following moderate exercise in patients with chronic fatigue syndrome (CFS) and that its postexercise expression was positively correlated with severity of mental and physical fatigue [17]. In female patients with medication refractory depression, we recently observed dysregulated gene expression during a depressive episode for three other ATP-responsive ion channels, including the purinergic receptors P2RX7 and P2RY1, and the transient vanilloid receptor TRPV1 [42]. Currently, studies are underway to determine if metabolite detecting receptor expression is altered in patients with depression as well as in those with CFS following moderate exercise. If gene expression changes underlie exercise-induced pain and fatigue symptoms, it may underlie depressive symptoms and therefore provide potential treatment targets.

### 4.4. Implications and Need for Future Research

Blood-based gene expression has a possible future in identification of dysfunction underlying disease and of novel treatment targets. In this pilot study examining 9 genes, gene expression differences following both ECT and ISO treatment were noted in patients with depression as well as pretreatment differences compared to controls. These preliminary results support the involvement of neuroimmune pathways in depression and treatment effects. This study had limited statistical power due to its small sample size, limited gene array, and several other limitations that can only be effectively addressed by subsequent research.

First, we only examined patients with medication refractory depression and we combined BPD and MDD patients. Thus even for the three mRNAs that showed trait-like differences between DD patients and controls, our study cannot determine whether these are biomarkers that distinguish medication refractory from medication responsive patients or based on depression diagnosis. Future studies including both types of DD patients are needed to address this issue. There clearly are many subtypes of depression, with unique gene expression patterns both before and following treatment. We previously reported on gene expression differences between female patients with MDD and BPD after controlling for medication use and depression severity [42]. Because our sample size was small, our findings may not generalize to the larger population of patients with refractory depression. It is hoped that future studies may be able to use gene expression to identify depression subtypes that match clinical observations.

Secondly, we report here on gene expression 24–48 hours following treatment. In some additional data obtained from 16 of these 22 DD patients who were also examined 4 weeks following end of treatment, there was continued dysregulation in DBI, ADRA2A, and ASIC3 despite generally good maintenance of depressive symptom improvement. This further reinforces our preliminary interpretation that dysregulation in these pathways may be trait-like characteristic in patients with medication refractory depression that could underlie their susceptibility to symptom relapse. In contrast, NR3C1 levels had returned to control levels after 4 weeks, suggesting that treatment-related changes in this biomarker may be time-dependent and unstable. Further studies are necessary to understand the relationship between gene expression, symptom presentation, and treatment response.

Thirdly, in our study we did not have any control subjects that were currently taking antidepressants or other mood-altering medications. It is therefore possible that differences in gene expression are the result of concurrent medication use. In fact, previous research suggests that antidepressants [1, 78, 79], anticonvulsants [42, 80], and antipsychotics [81] can have effects on gene expression and protein production. Therefore, medication use should continue to be examined for potential effects in gene expression studies, and researchers should consider comparisons between individuals on these same medications who are not currently experiencing a depressive episode.

Finally, this study only examined 9 genes in a candidate-driven study. We recently completed another study examining a diverse panel of 28 genes during a depressive episode and found further support for pretreatment dysregulation in inflammatory pathways as well as members of metabolic receptors, such as purinergic and vanilloid receptors [42]. Future studies with larger gene expression arrays and with larger subgroups of patients (including both medication-responsive and refractory bipolar and unipolar depression) is strongly encouraged to validate our preliminary findings.

## Figures and Tables

**Table 1:** Demographic and medical data for controls (CON) and depressive disorder (DD) patients at pretreatment.

	CON	ECT	ISO	All DD (ECT + ISO)
Number of subjects	*n* = 17	*n* = 14	*n* = 8	*n* = 22

Age (years)	47.5 ± 3.15	40.9 ± 3.2	38.5 ± 4.0	40.0 ± 2.3^a^
Gender: male/female	6/11	9/5	5/3	14/8
Diagnosis: MDD/BPD	—	11/3	6/2	17/5
Inpatient/outpatient	—	7/7	0/8	7/15
Number of treatments	—	9.9 ± 0.4	9.6 ± 0.4	9.8 ± 0.3
HRSD pretreatment	—	35.6 ± 1.8	26.6 ± 1.4	32.3 ± 1.5
HRSD posttreatment	—	12.7 ± 2.5^b^	12.5 ± 3.6^b^	12.6 ± 2.0^b^
QIDS pretreatment	—	18.7 ± 1.2	17.0 ± 0.8	18.0 ± 0.8
QIDS posttreatment	—	13.3 ± 1.3^c^	9.9 ± 1.4^c^	11.8 ± 1.0^c^
No medication	17	2	2	4
Any antidepressants (classes below)	0	11 (78.5%)	5 (62.5%)	16 (72.7%)
SSRIs	0	5	2	7
NRI	0	1	0	1
SNRI	0	6	3	9
Tricyclic	0	0	0	0
Anticonvulsants (none, stopped, continued)	0	4/4/6	4/2/2	8/6/8
Antipsychotics	0	4	5	9

^a^Age was marginally lower in All DD compared to CON group (*P* = 0.06), and thus was included as a covariate in all subsequent analyses where it was related to the outcome measure (at least *P* < 0.10).

^b^HRSD was significantly decreased at Post compared to Pre (ECT *P* < 0.001, ISO *P* < 0.01).

^c^QIDS was significantly decreased at Post compared to Pre (ECT *P* = 0.0046, ISO *P* = 0.0011).

SSRI (selective serotonin reuptake inhibitor), NRI (norepinephrine reuptake inhibitor), SNRI (mixed serotonin-norepinephrine reuptake inhibitor).

**Table 2:** Pretreatment gene expression in controls (CON), ECT, and ISO Groups.

Gene	CON (*n* = 17)	ECT (*n* = 14)	ISO (*n* = 8)	CON versus DD (ECT + ISO) *P* value	CON versus ECT *P* value	CON versus ISO *P* value	ECT versus ISO *P* value
ADRA2A*	5.26*E* − 03	2.12*E* − 03	1.91*E* − 03	0.0013	0.001	0.0285	0.541
	±1.25*E* − 04	±5.27*E* − 04	±4.81*E* − 04				

ASIC1	7.98*E* − 04	1.02*E* − 03	1.01*E* − 03	0.367	0.303	0.137	0.518
	±1.08*E* − 04	±2.03*E* − 04	±1.52*E* − 04				

ASIC3	8.32*E* − 03	6.01*E* − 03	5.54*E* − 03	0.003	0.0055	0.0065	0.684
	±7.39*E* − 04	±4.63*E* − 04	±3.52*E* − 04				

DBI	3.96*E* − 03	5.59*E* − 03	5.60*E* − 03	0.0004	0.001	0.003	0.979
	±2.99*E* − 04	±3.84*E* − 04	±5.85*E* − 04				

DRD4	1.84*E* − 03	2.26*E* − 03	2.29*E* − 03	0.927	0.466	0.476	0.992
	±2.54*E* − 04	±5.22*E* − 04	±5.92*E* − 04				

IL10	5.70*E* − 03	1.22*E* − 02	1.25*E* − 02	0.022	0.024	0.035	0.910
	±7.78*E* − 04	±4.36*E* − 03	±3.92*E* − 03				

NR3C1	7.90*E* − 01	7.05*E* − 01	7.15*E* − 01	0.465	0.285	0.255	0.860
	±7.40*E* − 02	±5.39*E* − 02	±1.10*E* − 01				

SULT1A1	1.44*E* − 03	1.56*E* − 03	1.26*E* − 03	0.778	0.196	0.254	0.195
	±1.20*E* − 04	±1.44*E* − 04	±1.20*E* − 04				

VEGFA	7.53*E* − 02	8.44*E* − 02	7.02*E* − 02	0.971	0.301	0.193	0.217
	±5.49*E* − 03	±9.71*E* − 03	±1.31*E* − 03				

Data shown as means ± SE and depict raw data; ANOVA statistics and contrasts used log-transformed data.

Age and male covariates were not significant and not included.

*Includes age as covariate.

Expressed in scientific notation units where *E* + 01 = ×10, *E* + 00 = ×1, *E* − 01 = ×0.1, *E* − 02 = ×0.01, and so forth.

**Table 3:** Gene expression for all 9 target genes at pretreatment versus posttreatment: means and ANCOVA outcomes for effects of treatment group (ECT versus ISO), sample time (pre- versus posttreatment) and group × time interactions.

Gene	ECT pre	ECT post	ISO pre	ISO post	Group *P* value	Time *P* value	Group × time *P* value
ADRA2A	2.26*E* − 03	3.21*E* − 03	1.90*E* − 03	2.46*E* − 03	0.731	0.430	0.137
	±6.26*E* − 04	±8.12*E* − 04	±5.70*E* − 04	±1.29*E* − 03			

ASIC1	9.46*E* − 04	1.24*E* − 03	1.01*E* − 03	1.12*E* − 03	0.995	0.390	0.162
	±2.17*E* − 04	±3.04*E* − 04	±1.52*E* − 04	±1.99*E* − 04			

ASIC3	6.25*E* − 03	6.60*E* − 03	5.54*E* − 03	6.40*E* − 03	0.925	0.187	0.412
	±5.16*E* − 04	±5.87*E* − 04	±3.52*E* − 04	±9.60*E* − 04			

DBI	5.49*E* − 03	5.39*E* − 03	5.67*E* − 03	5.62*E* − 03	0.904	0.492	0.436
	±4.60*E* − 04	±2.31*E* − 04	±6.70*E* − 04	±7.11*E* − 04			

DRD4	2.23*E* − 03	1.68*E* − 03	2.29*E* − 03	1.69*E* − 03	0.874	0.035	0.341
	±5.63*E* − 04	±4.61*E* − 04	±5.92*E* − 04	±4.56*E* − 04			

IL10	1.31*E* − 02	8.08*E* − 03	1.38*E* − 02	1.31*E* − 02	0.687	0.025	0.133
	±5.69*E* − 03	±2.73*E* − 03	±4.26*E* − 03	±4.16*E* − 03			

NR3C1	6.83*E* − 01	4.90*E* − 01	7.44*E* − 01	5.63*E* − 01	0.953	0.00015	0.389
	±7.44*E* − 02	±4.48*E* − 02	±1.22*E* − 01	±8.88*E* − 02			

SULT1A1	1.64*E* − 03	1.43*E* − 03	1.29*E* − 03	1.14*E* − 03	0.177	0.041	0.343
	±1.76*E* − 04	±1.61*E* − 04	±1.36*E* − 04	±6.24*E* − 05			

VEGFA	9.03*E* − 02	8.15*E* − 02	7.02*E* − 02	8.81*E* − 02	0.977	0.295	0.067
	±1.04*E* − 02	±1.30*E* − 02	±1.31*E* − 02	±8.65*E* − 03			

Data shown as means ± SE and depict raw data; ANCOVA used log-transformed data.

Expressed in scientific notation units, where *E* + 01 = ×10, *E* + 00 = ×1, *E* − 01 = ×0.1, *E* − 02 = ×0.01, and so forth.
